# Enhancing breast cancer detection with AI for early diagnosis and recurrence prediction

**DOI:** 10.18632/oncoscience.660

**Published:** 2026-05-19

**Authors:** Sidney Andre, Swarda Bandiwadekar, Mrunalini Pattarkine

**Affiliations:** ^1^Department of Biotechnology, Harrisburg University of Science and Technology, Harrisburg, PA 17101, USA

**Keywords:** AI in breast cancer screening modalities, early detection, artificial intelligence (AI), recurrence prediction, breast imaging

## Abstract

Breast cancer remains one of the leading causes of death among women worldwide, early detection is critical for improving survival. Conventional screening modalities such as mammography, MRI, biopsy, and ultrasound have been pivotal for patient care, but face limitations, including human variability, interpretive errors, operator dependence, and high rates of false positives and negatives which can lead to unnecessary treatment and financial distress. To overcome these challenges, a more accurate and predictive diagnostic tool is needed. This article compares traditional breast cancer screening methods with AI-integrated approaches demonstrating how artificial intelligence enhances diagnostic precision, efficiency, and predictive capability. AI-assisted mammography has been shown to detect 29% more cancers than traditional mammography without increasing false positives and reduces radiologist reading time by 40%. AI-assisted 3D digital breast tomosynthesis detects 1.6 additional cancers per 1,000 screenings and reduces recall rates by 2.2% compared to 2D mammography. In MRI, AI predicted breast cancer development up to one year in advance and correctly localize future cancer sites in 57% of cases. In ultrasound, AI significantly improves diagnostic accuracy, particularly for less experienced radiologists. In biopsy, AI-enabled digital assays for recurrence prediction achieve higher predictive accuracy, with pathologists using AI being 62% faster and 72% more accurate. Integrating AI into breast cancer redefines diagnostic excellence, shifting breast cancer management toward a more proactive, precise, and patient-centered approach. Future research should focus on external validation of AI models across larger and diverse populations, cost-effectiveness to ensure equitable access and ethical consideration to ensure responsible clinical adoption.

## INTRODUCTION

Breast cancer is one of the leading causes of death among women worldwide. It originates from the uncontrolled growth of cancer cells within breast tissue. Although breast cancer predominantly affects women, men can also develop the disease, albeit at much lower rates. According to the World Health Organization, approximately two million women are diagnosed with breast cancer globally each year, and about 600,000 women have died from breast cancer since 2020 [1]. These statistics underscore breast cancer as a major global health issue and highlight the need for continued investigation. Survival outcomes are strongly influenced by early detection, accurate diagnosis, access to qualified healthcare personnel, and advanced treatment technologies [2]. As a result, high-income and more developed regions are often able to detect breast cancer earlier due to greater access to these resources, while low-income regions may face limited availability, leading to higher breast cancer-related mortality [3].

A meta-analysis focused on women between the ages of 20 and 49 demonstrated that breast cancer mortality rates vary significantly by region: 10.2 per 100,000 women in wealthy regions, 15.5 per 100,000 in middle-income regions, and 20.3 per 100,000 in low-income regions, the highest among the three groups [3]. According to the Centers for Disease Control and Prevention, women are encouraged to begin annual breast cancer screening at age 40 to mitigate risk [4, 5]. Several screening modalities are available, including mammography, magnetic resonance imaging (MRI), ultrasound, and, when indicated, biopsy.

A study published by Harvard Medical School titled “The Mammography Dilemma” suggests that mammography alone reduces breast cancer mortality by 19%, and that women who undergo annual screening have a higher chance of survival [6, 7]. However, these methods are not without limitations and may contribute to adverse outcomes when misdiagnoses occur. Mammography, for example, is prone to false-positive and false-negative results and is less effective in patients with dense breast tissue [6].

Ultrasound is highly operator-dependent and has lower sensitivity, increasing the likelihood that cancer may be missed, particularly by less experienced clinicians. Additionally, while MRI offers high-resolution imaging, it is extremely costly and associated with high false-positive rates. Biopsy procedures are invasive, time-consuming, and expensive [8].

In the aforementioned Harvard Medical School study, approximately 19% of detected cancers were cases that would never become clinically significant, leading to unnecessary treatments that occur more frequently than they should. Among women who undergo annual breast cancer screening, more than 50% experience at least one false-positive result, and approximately 20% of these women undergo unnecessary biopsies, costing thousands of dollars and exposing them to potential health risks [6]. Given these significant limitations of current breast cancer screening methodologies, recent advancements in artificial intelligence present promising opportunities to improve both the accuracy and efficiency of traditional screening approaches [6]. AI-driven tools have demonstrated the ability to identify subtle patterns in imaging data, reduce diagnostic errors, and support radiologists and clinicians in making timely and accurate decisions.

For instance, a randomized clinical vignette study published in 2024 by Goh et al. in JAMA Network Open found that ChatGPT-4, a large language model, achieved diagnostic accuracy comparable to that of top-performing physicians while significantly improving efficiency in clinical decision-making [9]. These findings highlight the potential of artificial intelligence to complement human expertise and improve diagnostic outcomes. This review article aims to explore how AI can be integrated into existing breast cancer screening methods to enhance early detection, improve diagnostic accuracy, and predict recurrence, thereby reducing mortality rates and improving patient outcomes. To better understand how the integration of AI can be transformative, it is first essential to examine how breast cancer develops and progresses.

## REVIEW METHODOLOGY

**Figure 1:**
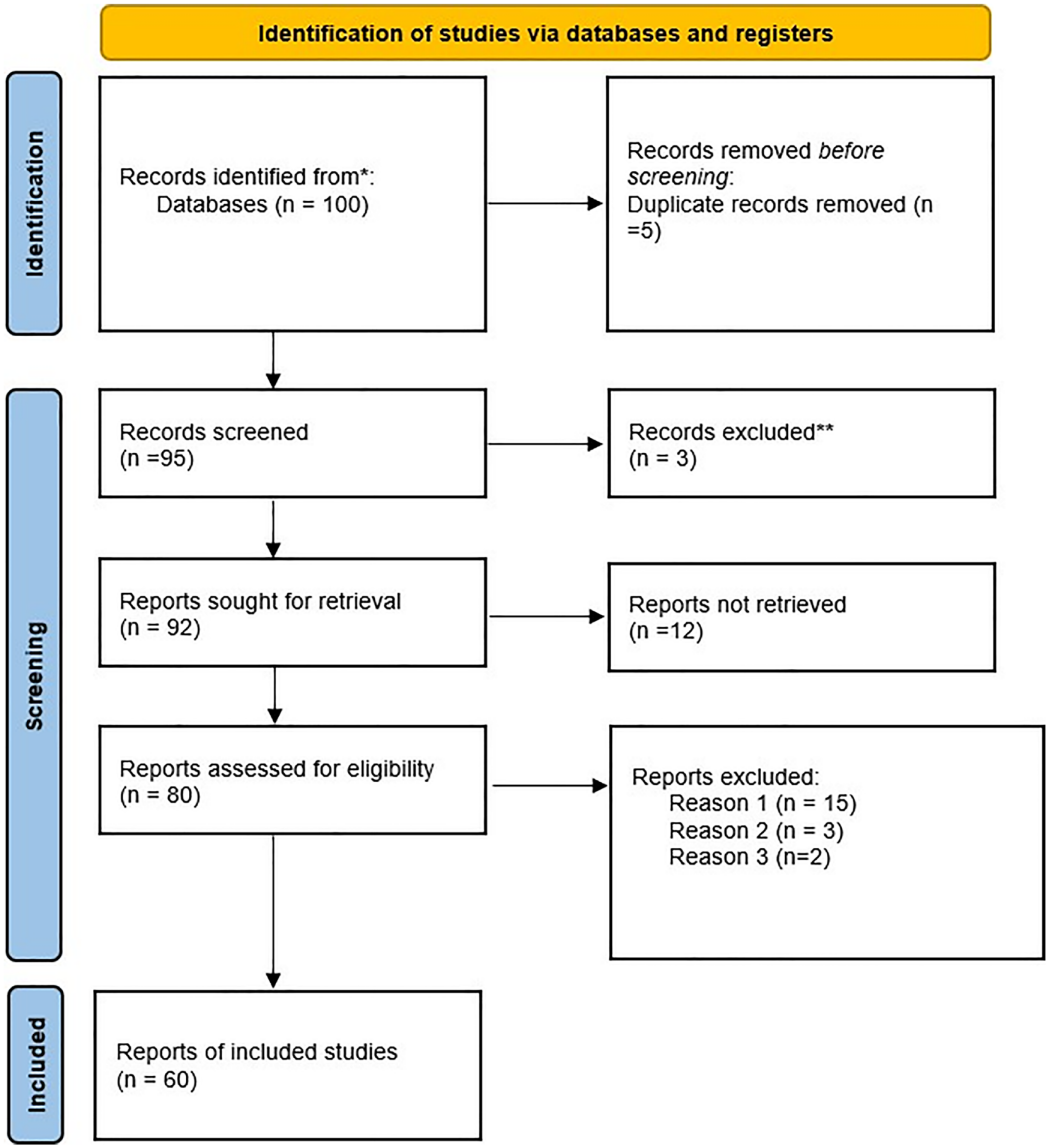
Prisma flow diagram [11].

To successfully conduct this review, PRISMA scoping review guidelines were followed (see Figure 1) [10, 11].

### Used research databases

PubMed, Scopus, ScienceDirect, ResearchGate, Google Scholar, Web of Science, JAMA Network Open, Nature, and other peer-reviewed journal articles.

### Keywords used

AI in Breast cancer screening modalities, Early detection, Artificial intelligence (AI), Recurrence prediction, Brest imaging.

### Time

Studies published between 2006 and 2025 were considered and included in this review.

### Inclusion criteria

Journal articles focused on traditional cancer screening methods, peer review articles focused on the integration of AI into traditional cancer screening methods, studies that have shown successful integration of AI in medicine, studies conducted across the world.

### Exclusion criteria

Articles with no relevant information, duplicated studies, non-English studies.

### Triage process

One hundred articles were reviewed, sixty articles were included based on relevancy and overall quality, and these included six case studies.

### Data handling

No new data was generated throughout this review; findings were synthesized from articles published articles reviewed in this study. An AI tool; OpenAI ChatGPT, was used only to assist with text and theme organization during scoping and articles selections within the research timeframe September - December 2025). No inclusion criteria, exclusion criteria, data extraction or quantitative analysis were automated. All Prompts and outputs were reviewed and edited by the authors for accuracy and attribution to meet the standards established by the international Committee of Medical Journal Editors [12]. Model access details are described in Appendix 1- AI Use Disclosure.

## BREAST CANCER OVERVIEW

**Figure 2:**
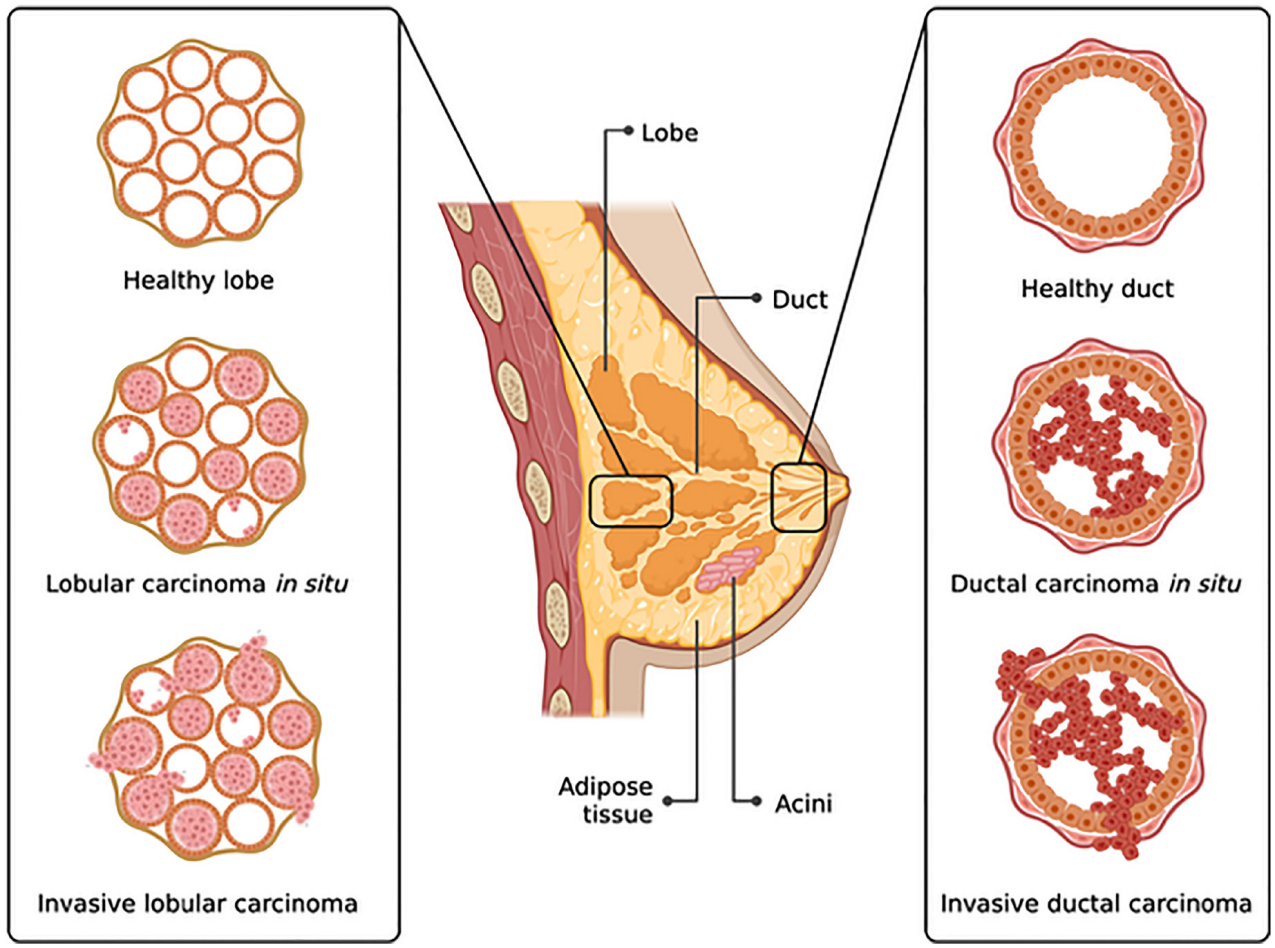
Ductal and lobular breast cancer overview [15].

Breast cancer can originate in the ducts or lobules of the breast. These cancers may be classified as *in situ*, which are generally considered noninvasive, or invasive, which are potentially life-threatening [13, 14]. Lobular carcinoma is a type of cancer that begins in the lobules, the regions where milk is produced [13]. In this condition, epithelial cells mutate and begin to grow uncontrollably, often without forming distinct lumps, making the disease difficult to detect in some cases. Ductal carcinoma, on the other hand, begins in the ducts, which are the pathways that transport milk to the nipple [13]. Figure 2 [15] illustrates the main anatomical parts of the breast and highlights the differences between healthy and diseased breast tissue.

The epithelial lining cells responsible for carrying milk through the ducts can undergo mutations and begin to grow uncontrollably, eventually accumulating within the duct. If not detected early, the cancer can spread to nearby tissues. Invasive ductal carcinoma (IDC) is the most common type of breast cancer, accounting for approximately 70–80% of diagnosed cases [4]. Invasive lobular carcinoma (ILC) is the second most common type, representing about 10–15% of diagnosed cases [4]. Breast cancers are further divided into subtypes based on the presence of estrogen and progesterone hormone receptors and the level of human epidermal growth factor receptor 2 (HER2) protein expression. These receptors allow breast cancer cells to use specific hormones to proliferate, while HER2 protein also promotes cancer growth. Detection of these biomarkers helps guide treatment decisions and assess the likelihood of cancer recurrence [4, 16]. A cancer lacking all these receptors or biomarkers is known as triple-negative breast cancer, which is considered difficult to treat because it does not respond to hormone-based or HER2-targeted therapies [17]. According to the CDC, breast cancer risk increases with age, with most diagnoses occurring in women over the age of 40, as women under 40 have a lower risk of developing breast cancer [4]. In the general population, the lifetime risk of developing breast cancer is approximately 12–13%, according to the National Cancer Institute [18]. Factors such as genetic mutations, particularly in BRCA1 and BRCA2, significantly increase risk. These genes function as tumor suppressors, and when harmful mutations occur, the likelihood of breast cancer rises substantially [19]. Inheriting these mutated genes further increases cancer risk. Additionally, individuals with a family history of breast cancer and women with dense breast tissue are at higher risk [18, 20]. Early stage detection is associated with survival outcomes; the balance of benefits and harms of screening varies by age and risk and should follow current guideline recommendations. Moreover, early detection often allows for treatment that is less invasive, less toxic, and less costly. According to the American Cancer Institute, women whose breast cancer is detected at an early stage have a survival rate of 99% [18]. There are multiple traditional methods available for early detection and prevention of breast cancer-related mortality, including mammography (two-dimensional and three-dimensional), magnetic resonance imaging (MRI), ultrasound, and biopsy.

## CONVENTIONAL BREAST CANCER SCREENING METHODS: MAMMOGRAPHY

**Figure 3:**
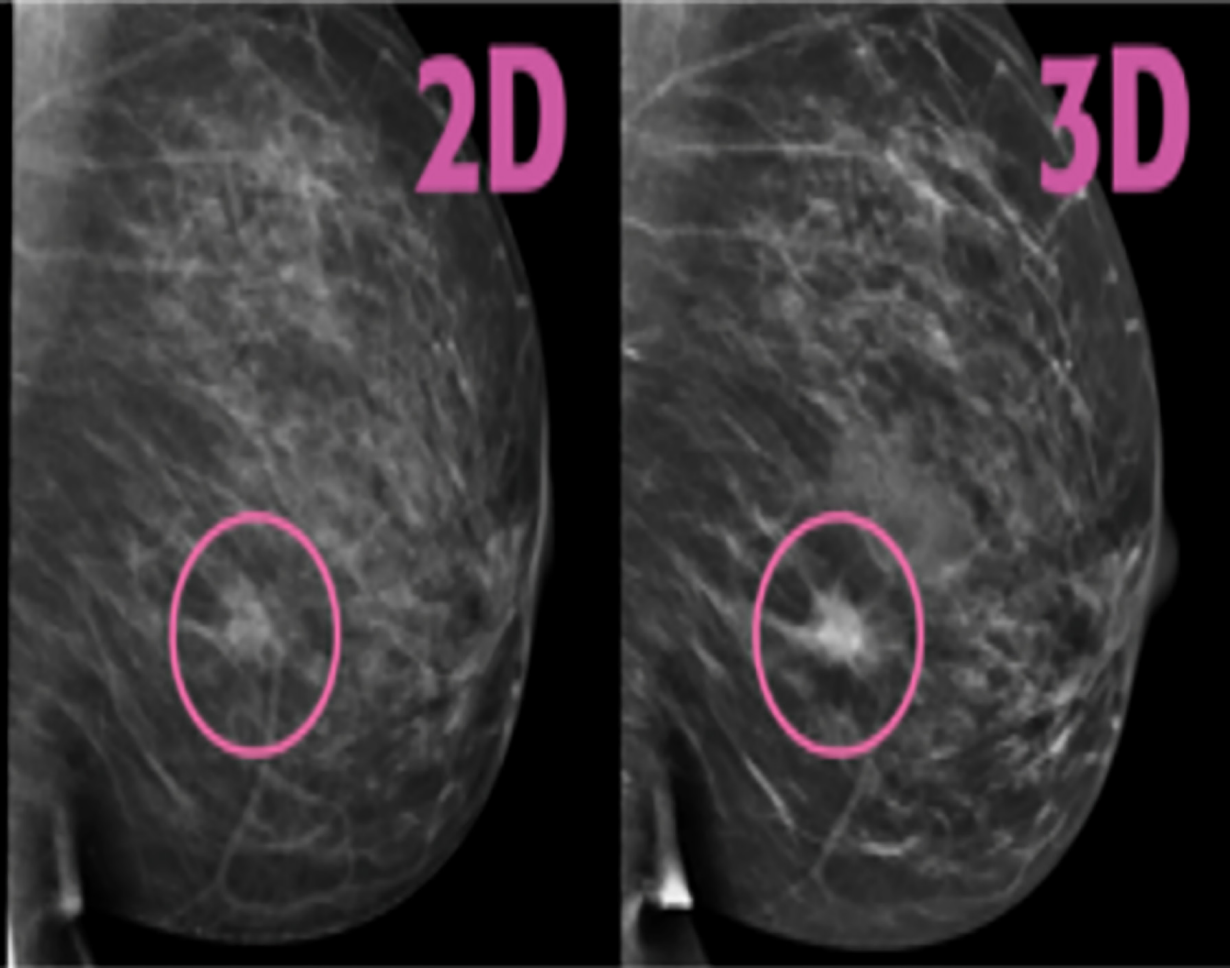
Comparison between 2D vs. 3D mammogram images [22].

A 2D mammogram is considered a traditional cancer screening method. It is a digital imaging technique that produces a flat, two-dimensional X-ray image of the breast. However, 2D mammograms often generate overlapping tissue images, which can make it difficult to detect small cancers [21]. Additionally, the X-ray machine produces only four images of each breast for analysis. In contrast, some consider the 3D mammogram to be revolutionary. In this method, the X-ray machine moves in a sweeping arc over the breast. This process is faster than 2D imaging and produces a series of images, up to 100 per breast, that capture detailed views of breast tissue, including small tumors [21]. Figure 3 [22] shows the differences between a 2D vs. 3D mammogram image.

A study by Marinovich et al. was conducted to compare 3D mammography with standard 2D mammography. This study utilized a large and inclusive database of relevant studies that reported cancer detection and recall rates. It combined results from multiple studies comparing 3D versus 2D mammography, allowing the authors to determine whether 3D mammography performed better, similarly, or worse than 2D mammography. A random-effects meta-analysis model was used to account for variability across studies, assuming that true effects differed between studies rather than a single fixed true effect. The meta-analysis found that 3D mammography detected an additional 1.6 cancers per 1,000 screenings compared to 2D mammography, a result supported by a 95% confidence interval [23]. Additionally, in studies using paired designs, cancer detection increased by approximately 2.4 per 1,000 screenings.

3D mammography also demonstrated a significant reduction in recall rates, with fewer women without cancer being called back for additional screening compared to 2D mammography-approximately 2.2% fewer recalls, or 2.2 fewer recalls per 100 screenings [23]. It is important to note that these improvements depended on factors such as screening frequency, breast density, and the type of screening used. Although this study is promising and shows significant improvement favoring 3D mammography, the use of a meta-analysis represents an observational and comparative approach rather than randomized controlled trials, which may introduce bias. Therefore, additional studies are needed to further assess whether the benefits of 3D mammography apply broadly to all women. Overall, however, 3D mammography has demonstrated superior performance by providing improved clarity and detail, increasing breast cancer detection by 27–50%, detecting cancer at earlier stages, and reducing the need for additional screening [24]. Due to limitations, particularly in detecting cancer in dense breasts, women are often recommended to undergo MRI for further evaluation.

## MAGNETIC RESONANCE IMAGING (MRI)

**Figure 4:**
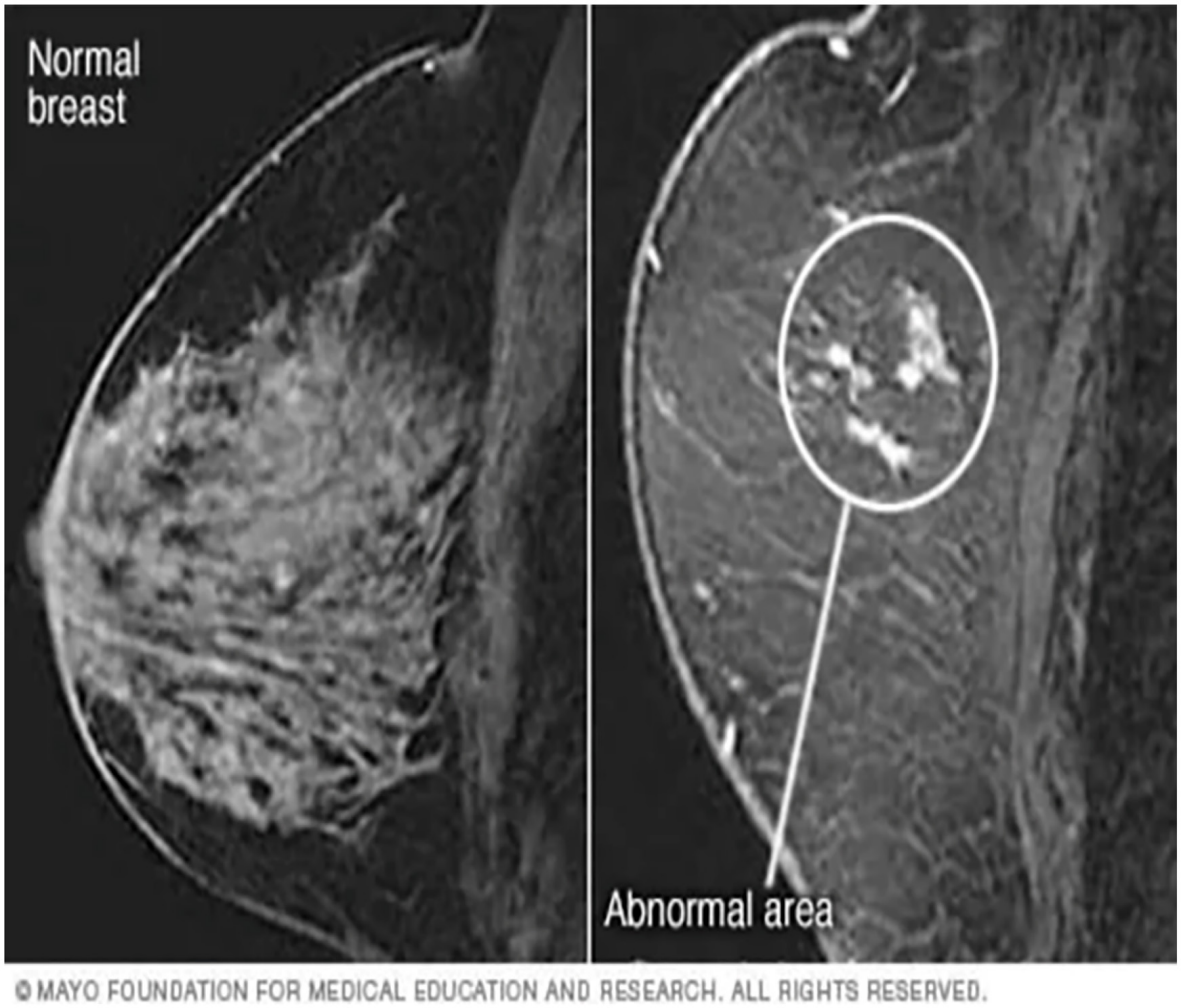
Difference between MRI scan of normal breast versus abnormal [28].

Magnetic Resonance Imaging (MRI) is one of the newer technologies used for breast cancer screening. MRI uses a strong magnetic field, radiofrequency pulses, and gradient magnetic fields to manipulate hydrogen atoms within the body, causing them to emit signals that are processed by a computer and converted into high-resolution images [25, 26]. During this process, a contrast agent is injected into a vein. As the breast is scanned through the MRI machine, tumors present in the breast will appear enhanced on the images [27]. Figure 4 [28] below shows how a normal breast versus an abnormal breast appear in an MRI scan.

Traditional MRI is typically used for women at higher risk for breast cancer [29]. High-risk women include those with BRCA1 or BRCA2 mutations, a family history of breast cancer, or prior exposure of the chest to radiation. MRI has high sensitivity, meaning it detects more cancers in high-risk women than mammography alone, particularly in women with dense breasts. Reported sensitivity ranges from 77–91%, indicating that fewer cases are missed when women undergo MRI screening [27]. Additionally, MRI identifies more cancers at earlier stages than other imaging methods. However, it has not been proven that MRI screening reduces breast cancer mortality more than mammography or other traditional screening modalities. MRI also has higher false-positive rates than other methods, which can lead to unnecessary additional testing or treatment. Furthermore, MRI is more expensive than mammography or ultrasound because it requires longer image acquisition times and highly trained personnel to operate the equipment [28]. Despite these limitations, MRI is still considered a reliable traditional modality for breast cancer detection, especially in women at substantial risk, due to its high sensitivity. Another modality that complements MRI is ultrasound, which also offers high sensitivity.

## ULTRASOUND

Ultrasound uses high-frequency sound waves that are transmitted into breast tissue, where the waves bounce back differently depending on tissue composition; these reflected waves are then used to generate images [30]. During breast cancer screening, ultrasound helps distinguish whether a lump is fluid-filled or solid, which aids in determining the likelihood of cancer. A study conducted by Berg et al. demonstrated that experienced breast radiologists showed variability in lesion detection and interpretation when performing breast ultrasound [31], highlighting the need for standardized imaging models and well-defined training processes to improve reproducibility and reliability of ultrasound-generated images.

Ultrasound is often used as a complementary screening modality for women with dense breasts, typically following mammography screening. Mammograms are known to miss cancers in dense breast tissue, making ultrasound a common recommendation in this population. However, ultrasound is also associated with a high rate of false positives, which can lead to unnecessary treatments and patient distress. Another limitation of traditional ultrasound is its dependence on operators; image quality and diagnostic accuracy rely heavily on the skill and experience of the radiologist or clinician interpreting the scans. As a result, variability in outcomes can occur depending on who analyzes the images.

A systematic review and meta-analysis conducted by researchers at the University of Tehran in Iran evaluated the sensitivity and specificity of traditional ultrasound for breast cancer detection compared to mammography in women at elevated risk. The researchers reviewed published studies up to 2023 from platforms including PubMed, Scopus, and others [32], following the PRISMA reporting guidelines. Of 19,022 articles initially identified, 28 studies were selected based on relevance and removal of duplicates [32]. For each study, data on true positives, true negatives, false positives, and false negatives were extracted. Statistical analyses were performed using STATA R software and hierarchical logistic-regression methods to calculate sensitivity and specificity with 95% confidence intervals [32].

Across all included studies, ultrasound demonstrated a pooled sensitivity of 87% (95% CI: 80–92%) and a specificity of 75% (95% CI: 61–84%) [32]. In comparison, mammography showed an estimated sensitivity of 78% (95% CI: 72–83%) and a specificity of 78% (95% CI: 66–86%) [32]. One limitation of this analysis is that it did not account for breast density or age differences, factors that could influence diagnostic performance. Overall, these findings suggest that ultrasound performs better than mammography in detecting true cancer cases in elevated-risk populations due to its higher sensitivity. However, the lower specificity indicates that more women may be referred for additional diagnostic procedures, such as biopsies, to confirm cancer presence and status.

## BIOPSY

Biopsy can also be used in breast cancer detection; it involves the removal of a sample of breast tissue for microscopic examination [33]. A pathologist analyzes the sample to confirm whether the tissue is benign or malignant and to identify the cancer type, such as lobular or ductal. Histopathological examination of biopsy tissue is the gold standard for confirming a breast cancer diagnosis because it provides direct visualization of cancer cells and allows determination of tumor type, stage, and receptor status [34]. When performed properly, biopsy can be a lifesaving procedure [35]. However, it is well established that this procedure is not easy for women, as it requires tissue removal that can cause pain, bleeding, and may lead to infection. Additionally, the diagnostic process may take days to weeks, and the associated costs are high, which can cause some women to avoid the procedure unless it is strongly recommended by their physicians.

A systematic review was conducted at McMaster University in Canada to compare two commonly used traditional biopsy techniques: fine needle aspiration cytology (FNAC) and core needle biopsy (CNB). This study also aimed to compare the sensitivity and specificity of these biopsy methods [36]. It included twelve prospective studies from multiple countries, including Brazil, Sweden, India, the United States, Pakistan, China, and the United Kingdom [36]. Data sources included articles from PubMed, EMBASE, MEDLINE, and the Cochrane Central Register of Controlled Trials up to February 2016. The selected articles compared the diagnostic accuracy of FNAC and CNB, with a total of 1,802 patients included across the twelve studies [36]. For data analysis, the researchers used forest plots to display sensitivity and specificity, assessed heterogeneity, and conducted pre-specified subgroup analyses (palpable vs. non-palpable lesions, publication year, and guidance type). CNB demonstrated a higher sensitivity of 87% (95% CI, 84–88%, I^2^) compared with FNAC at 74% (95% CI, 72–77%, I^2^ = 88.3%) [36]. The specificity of CNB and FNAC was comparable, at 98% (95% CI, 96–99%, I^2^) and 96% (95% CI, 94–98%, I^2^), respectively [36]. Additionally, subgroup analyses showed higher sensitivity for palpable lesions compared with non-palpable lesions, further supporting the reliability of biopsy in cancer detection [36]. Overall, this study indicates that both FNAC and CNB demonstrate good clinical performance. See Table 1 for the summary comparison among cancer screening modalities.

**Table 1:** Comparative strengths and weaknesses of conventional screening methods (Andre et al., 2025)

Method	What it is/Role	Advantages	Limitations	Best use
Mammography (Marinovich et al., 2018 and Lauby-Secretan et al., 2015) [37]	X-ray imaging of the breast.	Associated with reducing breast cancer mortality in women between the age of 50–79 Widely available Short exam time	Standard drops in dense breast. False Positives/recalls. May miss small lesions hidden by overlapping tissues.	Standard screening tool for average-risk women.
Ultrasound (Berg et al., 2006)	Uses sound waves to image breast tissue.	No radiation; Helps distinguish solid vs. cystic masses. Useful in dense breasts; Portable and low cost.	Highly operator dependent. High rate of false positive. Limited penetration in some tissues.	Supplemental screening, follow-up of suspicious findings
MRI (Houser et al., 2021)	Magnetic and radiofrequency imaging, often with contrast.	Extremely high sensitivity for detecting cancers in high-risk women. Less affected by breast density. Good at detecting lesions missed by mammography.	Higher cost; High rate of false positive. Longer scanning and interpretation time; Not widely available	Screening in high-risk women. Good for screening dense breast cancer.
Biopsy (Anglade et al., 2020)	Tissue sampling/histopathology.	Gold standard for definitive diagnosis; Confirms whether a lesion is benign or malignant; Guides treatment planning (stage, receptor status).	Invasive. Risk of complication (Bleed and infection). Patient discomfort and anxiety.	Diagnostic confirmation after imaging suggests a suspicious finding.

Table 1 shows the summary results of comparison among screening modalities highlighting their limitation and advantages. Despite the significant progress and advancements made in medicine to date, women continue to face challenges in the diagnostic process, one of which is misdiagnosis. This often leads to unnecessary treatments, mental health issues, financial distress, and, in extreme cases, death. As noted in a study published by Goh et al., artificial intelligence has a strong track record of complementing human expertise to deliver improved outcomes [9]. With the integration of artificial intelligence into precision medicine, many of the limitations faced by traditional cancer screening modalities may soon become a problem of the past.

## ARTIFICIAL INTELLIGENCE (AI) IN BREAST CANCER SCREENING

**Figure 5:**
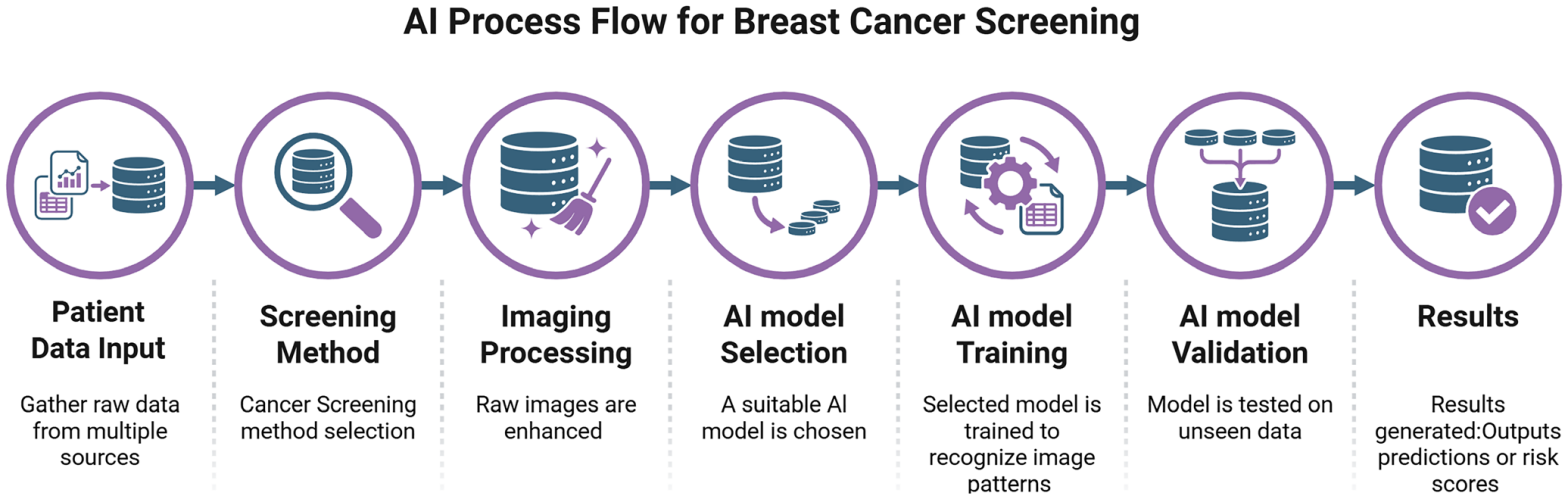
AI process flow for breast cancer screening [44].

AI plays a crucial role in delivering accurate results more rapidly than traditional cancer screening methods. AI provides detailed scanned images that highlight suspicious areas, including lesions that are small and difficult to detect [38]. It also analyzes shapes and patterns within the generated images to help determine the severity of detected lumps [39]. Additionally, AI identifies abnormal blood patterns and changes in blood flow that may indicate the presence of cancer [40]. In biopsies, AI analyzes tissue samples faster and more accurately than traditional microscopy. For instance, an algorithm called Rare Event Detection (RED), developed by the University of Southern California, automates the identification of rare cancer cells, including breast cancer cells, among millions of normal blood cells within minutes [41]. Furthermore, a study published by the Stanford School of Medicine showed that pathologists using AI were 62% faster than those who did not use AI and achieved a 72% higher accuracy rate compared with traditional microscopic examination [42]. These advancements demonstrate that AI has not only transformed breast cancer screening methods but has also significantly improved pathological workflows [43], as shown in Figure 5 [44].

In the first step, patient information such as age and family history of breast cancer is collected. This information helps radiologists and clinicians select or personalize an AI model to better meet individual patient needs. Following this step are the screening methods, in which the patient or, in some cases, the radiologist chooses the most appropriate modality. Factors such as cost, time, and health conditions may influence this choice. Screening methods may include mammography, ultrasound, MRI, or biopsy [8, 45]. The images generated through these methods serve as the primary input for AI analysis. Once a screening method is selected and images are generated, the images are considered raw, necessitating further processing. During this step, images are enhanced, noise is reduced, contrast is adjusted, and suspicious regions of the breast are highlighted. This step is crucial because it prepares a high-quality, standardized input for AI analysis [8, 45].

Next, the AI model is selected on a case-by-case basis, as breast cancer presentations vary across patients. The model is chosen based on the type of data and diagnostic goal to ensure alignment with the complexity of the imaging data. Once selected, the model is trained using large datasets of labeled images with diagnoses initially determined by radiologists. The purpose of this step is to train the AI model to recognize patterns associated with benign and malignant findings [8, 45]. The model is then validated using unseen data to evaluate its accuracy, sensitivity, and specificity. This step is critical, as it ensures reliable performance prior to clinical deployment [8, 45].

The validation process provides a systematic approach to assessing the reliability, accuracy, and generalizability of the AI algorithm before its use in breast cancer screening, ensuring consistent performance across clinical scenarios. A typical validation process consists of three or more steps. First, a train-test split is performed, in which the dataset is divided into two parts: one used to train the model to learn diagnostic patterns, and the other used to test performance on new, unseen cases. This step is important to confirm that the model generalizes rather than memorizes the data [8]. Second, cross-validation is conducted. Instead of evaluating the model using a single split, the dataset is divided into *k* subsets, allowing the model to be trained and tested multiple times. This approach provides a more reliable assessment of performance by ensuring that all portions of the dataset are evaluated. Following these steps, hyperparameter tuning is performed, during which learning parameters are optimized to ensure accuracy and computational efficiency during analysis [8].

The biomarkers used to identify the presence of cancer vary across algorithms but commonly include tumor size, texture features, edge features, density patterns, symmetry, concavity and concave points, and fractal dimension [8]. Finally, the validated AI model is qualified and certified to analyze new patient images and generate risk score predictions. These outputs support radiologists in detecting breast cancer earlier and with greater accuracy. The following figure illustrates the workflow of AI-enhanced medical imaging in practice.

To better understand the impact of AI on traditional breast cancer screening modalities, a series of case studies from institutions around the world were reviewed.

## CASE STUDY I- AI IN MAMMOGRAPHY

Researchers in Sweden investigated whether integrating artificial intelligence with mammography could enhance early cancer detection. They employed an AI system, Transpara v1.7.0, with the goal of improving diagnostic accuracy and testing performance. The AI algorithm was validated to perform in-depth analyses and highlight image features on 2D mammograms. For cancer characterization, immunohistochemical biomarkers including estrogen receptor (ER), progesterone receptor (PR), human epidermal growth factor receptor 2 (HER2), and the Ki-67 proliferation index were used to identify triple-negative, HER2-positive, and luminal cases [46]. A randomized, controlled study was conducted involving approximately 105,934 women aged 40 to 80 [46]. Participants were divided into two groups based on the screening method: group 1 underwent assessment by a single radiologist assisted by AI, while group 2 was evaluated by two radiologists using the traditional approach. In this study, artificial intelligence was integrated to stratify patients by cancer risk and highlight suspicious regions through detailed image analysis. The screening protocol included two mammographic views, craniocaudal and mediolateral oblique, with an implant displacement view for patients with implants [46]. To ensure consistency, all mammograms were generated using the same imaging system, and the AI-assisted group specifically used Transpara v1.7.0 [46].

The results demonstrated that AI integration has the potential to improve overall screening outcomes. Group 1, which involved radiologist assessment assisted by AI, detected 29% more cancers than the traditional group 2 approach without an increase in false-positive rates. All detected cancers were at an early stage, suggesting that the algorithm functioned as intended. In addition, radiologist reading time was reduced by 40% with AI support, and false-positive rates were comparable between the AI-assisted and control groups at 1.5% and 1.4%, respectively, supporting the reliability of AI-assisted screening [46]. A detailed summary of the results of this study can be found [here]. This study represents a step forward in the integration of AI into precision medicine. Although approved by the Swedish Ethical Review Authority, the study has several limitations. All images were acquired from a single vendor and assessed using a single algorithm, which limits generalizability. Furthermore, despite the large study population, the lack of external validation and limited diversity in the sample population reduce its representativeness of a broader population [46]. No cost analysis was included, leaving the financial implications of widespread adoption uncertain.

The previously referenced study by Marinovich et al. evaluated the performance of AI-assisted 3D mammography image processing. This systematic review and meta-analysis was conducted between 2009 and 2017 across Europe and the United States and included approximately one million women aged 50 to 59. The 3D method, known as digital breast tomosynthesis (DBT), is a breast imaging technique that uses multiple low-dose X-ray images acquired from different angles and reconstructs them into thin slices for three-dimensional visualization [23]. The AI algorithms used in DBT analysis were validated and relied on morphological biomarkers, which describe the size, shape, and structure of organs and lesions to classify tumors as benign or malignant, as well as radiomic biomarkers, which capture quantitative imaging features such as intensity, texture, and heterogeneity that are not visible to the human eye. A key limitation of 2D mammography compared with 3D imaging is tissue overlap, which can obscure lesions and lead to missed diagnoses. DBT addresses this issue through algorithmic reconstruction and computer-aided detection to reduce tissue overlapping and improve lesion visibility [23]. As a result, 3D AI-assisted mammography outperformed 2D AI-assisted mammography, demonstrating a higher cancer detection rate. Specifically, 3D imaging detected 1.6 additional cancers per 1,000 screenings compared with 2D imaging, supported by a 95% confidence interval of 1.1 to 2.0 and a *p*-value less than 0.001, and was associated with fewer recalls, which decreased by 2.2% with a 95% confidence interval of −3.0 to −1.4 and a *p*-value less than 0.001 [23]. A detailed summary of the results of this study can be found [here]. Women with dense breast tissue particularly benefited from this approach, as the reduction in overlapping tissue improved lesion identification. However, the results in this subgroup were not sufficient to support a statistically significant conclusion.

## CASE STUDY II- AI IN MAGNET RESONANCE IMAGING

A retrospective study by Hirsch et al. focused on the development of an AI model to predict the early development of breast cancer up to one year in advance using MRI. Approximately 910 women in New York, USA, participated in the study, with ages ranging from 18 to 88 years. The study was conducted at Memorial Sloan Kettering Cancer Center in New York between 2002 and 2014, during which a total of 3,029 MRI scans were generated. This dataset included 115 cancers that developed within one year following a negative MRI and 4,965 MRIs that were classified as benign based on at least two years of follow-up imaging with BI-RADS ≤3 [47].

The AI algorithm employed was a two-dimensional convolutional neural network (CNN) that utilized sagittal MRI slices. CNNs are a class of neural networks commonly used for image recognition and processing due to their ability to identify complex patterns within images [48]. The biomarkers used by the model to flag the presence of cancer included subtle tissue changes and signal patterns in normal-appearing breast tissue. Indicators of cancer presence included mass shape, architectural distortion, and parenchymal background. Quantitative lesion features were derived from segmentation and parenchymal enhancement analysis, a detailed image analysis approach that divides images into multiple segments to facilitate identification of suspicious regions of the breast [47].

The CNN was optimized using 10-fold cross-validation, in which the model was repeatedly assessed on different subsets of the data to evaluate its ability to handle rare and previously unseen cases, thereby ensuring robust and reliable performance. In addition, the model was initially trained on similar MRI datasets and subsequently fine-tuned with new MRI scans to confirm its capacity to process images effectively. The dataset included MRI scans labeled as benign, potentially malignant, or neither. The role of AI was central, as it analyzed sagittal MRI slices, assigned risk scores, and predicted cancer location using automated segmentation and registration tools [47].

The study noted a low prevalence of cancer within the screening population, which posed challenges for cancer detection and model training due to the large number of parameters involved. To address this limitation, the investigators leveraged an existing internal model that had been trained and validated on a dataset of 11,000 patients. For the top 10% of patients with the highest predicted risk, the system was retrained to optimize performance. In each fold of the cross-validation process, 90% of MRI scans were used for training and 10% were used for testing [47].

The results demonstrated the effectiveness of the model, with predictive accuracy measured by the area under the receiver operating characteristic curve (AUC-ROC) of 0.72 (95% CI: 0.67–0.76), indicating strong performance. Additionally, the model correctly identified cancer locations in 57% of cases, with 66 of 115 cancer locations accurately localized [47]. Missed cases included lesions as small as 0.5 cm, and agreement between AI and radiologists occurred in 47% of cases [47]. Small breast tumors, especially less than 1 cm are hard to detect by MRI and AI models because of their size, they can easily blend with normal breast tissue and may be lost when images are compressed or analyzed slice by slice. To address this issue, new AI models such as YOLO and long short-term memory (LSTM) networks are being developed to automatically detect small breast lesions on MRI and reduce false positives, and this will help radiologists to focus on the most suspicious areas [49].

Furthermore, MRI scans ranked within the top 10% of highest risk by the AI could have increased early detection by up to 30%, as 35 of the 115 cancers that developed within one year were flagged as high risk [47]. The detailed result summary can be found [here].

Despite its promising performance, the study employed a retrospective design, meaning that results were derived solely from historical data at a single cancer center, which limits generalizability. The model lacks external validation, and therefore real-world performance cannot be confirmed. Additionally, only one imaging plane was included, namely sagittal MRI scans. To more comprehensively evaluate the model, other imaging orientations such as axial scans and multi-site datasets should be incorporated. No cost-benefit analysis was included, leaving the financial implications of implementation and patient impact unknown. The model is not FDA approved, and the authors emphasize that it serves as a proof of concept. Nevertheless, the retrospective study was conducted under Institutional Review Board approval, with HIPAA compliance and a waiver of informed consent.

## CASE STUDY III- AI IN ULTRASOUND

This single-center retrospective study was conducted at Yeditepe University School of Medicine in Istanbul, Turkey, between September 2022 and August 2023 [50]. The study focused on integrating an artificial intelligence system, specifically the Koios Decision Support System, into conventional ultrasound imaging to assist radiologists in the detection of breast cancer. As a retrospective, single-center study, it utilized existing data from a single institutional database. Approximately 70 women aged 32 to 87 years with suspicious breast lesions were included; among them, 53 lesions were malignant and 17 were benign, as determined by biopsy [50]. A GE LOGIQ E9 ultrasound system was used in conjunction with the Koios Decision Support System, Breast Imaging Reporting and Data System (BI-RADS) classification, and shear wave elastography (SWE) to assess lesion stiffness [50].

The deep learning decision support model was trained to segment breast lesions on ultrasound images and extract morphological features such as shape, margin, and echogenicity [50]. Transverse and sagittal grayscale ultrasound images, along with SWE images, were evaluated by an experienced radiologist with approximately 15 years of experience, who assessed the lesions in real time. A less experienced radiologist with approximately one year of experience evaluated the lesions with assistance from the AI system [50]. The AI system classified lesions into BI-RADS categories based on image-derived features, after which the radiologist reanalyzed the images to reach a conclusion.

Statistical analysis included receiver operating characteristic (ROC) curve analysis and calculation of the area under the curve (AUC), as well as sensitivity, positive predictive value, negative predictive value, and specificity. In addition, SWE classified lesions with stiffness values below 20 kPa as benign and values greater than 138 kPa as malignant. Table 2 summarizes the study findings, highlighting differences in diagnostic performance between an experienced radiologist and the less experienced radiologist assisted by the AI system.

**Table 2:** Summary of the study result (Sidney Andre et al., 2025)

Group	AUC	Sensitivity (%)	Specificity (%)	Accuracy (%)
Experienced Radiologist (Çelebi et al., 2025)	0.888	98.1	58.8	88.6
AI System (Çelebi et al., 2025)	0.693	92.5	35.3	78.6
Less experienced Radiologist (Çelebi et al., 2025)	0.512	84.9	17.6	68.6
Less experienced Radiologist + AI (Çelebi et al., 2025)	0.655	90.6	17.6	72.9
Less experienced Radiologist + AI+ Elasticity (Çelebi et al., 2025)	0.679	92.5	23.5	75.7

Detailed summary results can be found [here]. AI played a crucial role in achieving these results, particularly for less experienced radiologists, as shown in the table above. Some participating radiologists were less experienced or not formally validated. This study demonstrated that experience is still required to correctly predict or diagnose cancer. Artificial intelligence can fill certain gaps that radiologists especially less experienced may be unable to address and can also support more accurate and faster image analysis. A key limitation of this study is the small sample size, with only 70 women included. A larger population would be necessary to more robustly validate this approach. AI assistance improved the performance of less experienced readers but did not match an experienced radiologist as seen on Table 2. Specificity remained modest to low indicating that AI systems complement but do not replace clinical expertise. A more diverse and slightly larger group of radiologists could potentially influence diagnostic outcomes. This study was conducted with IRB approval and ethics clearance and was approved by the Istanbul Bilgi University Ethics Committee with informed consent. HIPAA compliance was strictly maintained by ensuring patient anonymity [50]. No FDA or CE certification for routine clinical use was granted either before or after studying. Although the Koios DS system is commercially available, it requires regulatory approval before integration into clinical workflows. No cost analysis was performed; however, the authors suggest that most costs would be associated with purchasing Koios DS, obtaining a usage license, and system integration [50]. Given the low specificity of the model, patients may also require additional screening procedures, such as biopsy, which are associated with increased cost.

## CASE STUDY IV- AI IN BIOPSY

This retrospective clinical and validation study was conducted to assess the risk levels of cancer recurrence and took place at Mount Sinai Hospital in New York. The goal was to develop and validate PDxBr, a digital breast cancer test that integrates artificial intelligence and clinical data to predict breast cancer recurrence in women with a prior diagnosis and a family history of the disease [51]. The study included 2,075 women aged 23 years and older, including women with a history of invasive ductal breast cancer [51]. The AI model used 80% of the collected data for training and validation, while the remaining 20% was used to conduct the final analysis to ensure reliability and applicability. Importantly, the AI model analyzed H&E-stained images to generate a patient-specific recurrence risk score ranging from 0 to 100. The analysis incorporated features such as age, tumor size, and lymph node status [51]. Approximately 800 images were generated per patient over a six-year period, which were subsequently reduced to 40 images that served as key determinants for diagnosis. These selected images were combined with clinical features including age, tumor size, family history, cancer stage, and lymph node status. Support regression for censored data was used to generate the final risk score on a scale of 0 to 100 [51].

Finally, women with a risk score of 58 or higher were classified as having substantial risk, whereas women with scores below 58 were classified as having minimal risk. The AI model was also able to identify histopathologic patterns related to tubule formation and tumor-infiltrating lymphocytes [51]. Table 3 presents the results obtained in this study.

**Table 3:** Summary results (Sidney Andre et al., 2025)

Cohort	AUC	Sensitivity	Specificity	Hazard ratio for recurrence
Training (*n* =1559) (Fernandez et al., 2022)	0.78	0.72	0.77	5.5 (*p* < 0.001)
Validation (*n* = 516). (Fernandez et al., 2022)	0.75	0.60	0.77	4.4 (*p* < 0.001)

Table 3 above summarizes the performance of the AI model, a comprehensive and detailed result summary can be found [here]. The model was able to process images at a faster rate than the human eye and demonstrated a higher accuracy of 0.75 compared with the accuracy of traditional models, which was 0.71. When combined with the Oncotype DX recurrence score, the predictive accuracy improved to 0.76 [51]. The model exhibited high sensitivity and specificity, indicating that in most cases women received the correct diagnosis. Furthermore, the hazard ratio compares the likelihood of cancer recurrence between the high-risk and low-risk groups; a hazard ratio of 4.4 implies that women in the high-risk group are 4.4 times more likely to experience cancer recurrence within 6 years after remission. A summary of the detailed results of this study is provided elsewhere [51].

Although this study and model show promising results, further evaluation in larger and more diverse populations is necessary to ensure consistent performance across individuals of different ethnic backgrounds. The model was trained on a database from a single source, Mount Sinai Hospital, and therefore requires external validation before broader application. Follow-up was limited to 6 years, and long-term outcomes remain unknown. This study was approved by the institutional review board, with informed consent waived; however, the model is not FDA approved and is currently used as a research tool to provide insight into how AI may be used to advance precision diagnosis. No direct cost analysis was carried out, although additional costs related to AI software integration could potentially affect the overall cost of biopsy procedures.

## DISCUSSION

The integration of artificial intelligence into traditional breast cancer screening methods represents a transformative advancement in precision and diagnostic medicine, and the evidence presented in this review article clearly demonstrates that AI not only enhances the accuracy and speed of traditional imaging modalities but also effectively addresses critical challenges such as human variability, interpretive errors, and inefficiencies that have historically limited these screening approaches. Across mammography, MRI, biopsy, and ultrasound, artificial intelligence has demonstrated superior performance in detecting early-stage cancers, reducing false diagnoses, and significantly improving diagnostic precision, all of which are pivotal for improving survival outcomes. Traditional breast cancer screening methods, while having saved countless lives through early detection, are not without limitations. Mammography, for instance, struggles to detect cancer in dense breast tissue, leading to high rates of false positives and unnecessary recalls that can cause substantial patient distress and financial burden [23].

Magnetic resonance imaging offers high sensitivity, but it is costly and prone to false positives, which limits accessibility and may contribute to overtreatment [27]. Although considered the gold standard for definitive diagnosis, it is invasive, time-consuming, and expensive [35]. Ultrasound remains highly dependent on operator skill and experience, introducing variability in diagnostic quality [31]. These limitations underscore the need for intelligent systems that can alleviate workload, enhance accuracy, improve reproducibility, and increase efficiency without replacing human expertise. Table 4 summarizes the comparative analysis of AI-integrated cancer screening modalities and highlights the strengths, weaknesses, and optimal use scenarios for each modality.

**Table 4:** Comparative analysis: Conventional vs. AI-Enhanced screening (Sidney Andre et al., 2025)

Modality (conventional → AI-integrated)	Key performance change	Quantitative metrics	Practical benefit	Limitations
Mammography: 2D/radiologist-only → Radiologist + AI Transpara v1.7.0 (Hernström et al. 2025) [37]	AI + radiologist detected 29% more cancers vs. two radiologists. Reader time reduced.	+29% cancers detected; false-positive rate 1.5% (AI group) vs. 1.4% (control); reader time ~40% shorter with AI.	Substantially increases cancer detection while not increasing (meaningfully) false positives; reduces radiologist workload/time.	Single large, randomized study (Sweden); needs replication in other systems/populations
Mammography: 2D → 3D DBT (AI-assisted DBT > 2D) (Marinovich et al., 2018)	3D/DBT (with AI processing mentioned in paper) improves detection and reduces recalls compared with 2D.	+1.6 cancers per 1,000 screens (DBT vs. 2D; 95% CI reported); recalls reduced by ~2.2%.	Better lesion visibility (less tissue overlap) → higher detection, fewer unnecessary recalls.	Meta-analysis heterogeneity: effect may vary by breast density and screening frequency.
MRI: traditional reading → AI-assisted MRI (predictive CNN model) (Hirsch et al., 2024)	AI predicted near-term (up to 1 year) cancer risk from MRI with moderate discrimination.	AUC = 0.72 (95% CI 0.67–0.76) for individual MRIs; correct location prediction 57%; missed lesions as small as 0.5 cm	AI can flag high-risk patients and location earlier than human read alone; useful for risk stratification and targeted follow-up.	AUC indicates moderate performance; detection of small lesions remains limited. Dataset/Time and single-center aspects affect generalizability.
Ultrasound: operator-dependent radiologist → AI-assisted ultrasound (Koios Decision Support) (Çelebi et al. 2025)	AI improved diagnostic performance particularly for less-experienced readers; helped assign BI-RADS and improved ROC/AUC metrics in the study.	Study cohort *n* ≈ 70 (53 malignant, 17 benign). AI aided sensitivity/AUC improvements for less experienced readers (paper reports ROC/AUC and sensitivity/specificity analyses).	Reduces operator dependence; can raise accuracy of inexperienced readers toward expert level and standardize lesion characterization.	Small, single-center retrospective cohort (*n* = 70) → limited statistical power and external validity; overall specificity remained low (false positives).
Biopsy/Histopathology: pathologist read → AI-enabled digital assay (PDxBr) (Fernandez et al. 2022)	AI model produced higher predictive accuracy for recurrence risk and processed images faster than human read alone.	Accuracy/AUC: AI model 0.75 vs. traditional models 0.71; combined with Oncotype DX → 0.76. Hazard ratio for high vs. low AI score = 4.4 (higher risk group much more likely to recur).	Improves recurrence-risk stratification; augments pathology by highlighting morphological features and integrating clinical variables. Faster processing/consistency.	Needs larger, diverse external validation; risk of overfitting to development cohort; clinical impact (change in management) requires prospective testing.

The studies detailed in this review article demonstrate how AI integration directly addresses these limitations, often outperforming conventional cancer screening modalities. In mammography, AI has significantly improved diagnostic capabilities. A randomized, controlled study showed that AI-assisted mammography detected 29% more cancers than the traditional approach using two radiologists, without increasing false-positive rates [53]. Equally important, this integration reduced radiologist reading time by 40%. Additionally, the evolution from 2D to 3D mammography augmented with AI has shown meaningful benefits. An AI-assisted 3D digital breast tomosynthesis approach, reported in a meta-analysis, identified 1.6 additional cancers per 1,000 screenings compared to 2D mammography and reduced recall rates by approximately 2.2%. These findings demonstrate that AI can refine image interpretation and standardize diagnostic outcomes, particularly by mitigating tissue overlap, which is a common limitation of 2D mammography [23].

In magnetic resonance imaging, AI extends prognostic capabilities beyond traditional imaging. An AI model demonstrated the ability to predict the early development of breast cancer up to one year in advance, achieving a predictive accuracy with an AUC of 0.72. Furthermore, the model correctly localized future cancer sites in 57% of cases, a level of predictive performance not achievable with conventional MRI alone. By detecting subtle tissue changes and signal patterns that are often imperceptible to the human eye, AI transforms MRI from a purely diagnostic tool into a powerful prognostic modality, enabling advances in risk stratification and early intervention [45].

In ultrasound, AI plays a crucial role in standardizing diagnostics, particularly in a modality where operator dependence remains a major challenge. One study showed that integrating an AI system into traditional ultrasound workflows significantly improved diagnostic accuracy, especially among less-experienced radiologists. The group of less-experienced radiologists assisted by AI achieved an AUC of 0.66, compared with an AUC of 0.51 for less-experienced radiologists without AI support, bringing their performance closer to that of experienced radiologists, who achieved an AUC of 0.89 [50].

In biopsy and pathological assessment, AI implementation is revolutionizing clinical workflows. An AI-enabled digital breast cancer assay for recurrence prediction achieved a predictive accuracy of 0.75, surpassing traditional models that typically demonstrated an accuracy of 0.71. When combined with the Oncotype DX recurrence score, predictive accuracy further increased to 0.76. Beyond improvements in accuracy, AI models have demonstrated substantial gains in efficiency [53]. A study published by Stanford Medicine reported that pathologists using AI were 62% faster and achieved 72% higher accuracy compared with those relying solely on traditional microscopic examination [51]. Furthermore, literature has shown that liquid biopsy assays such as ctDNA (circulating tumor DNA) increasingly complement imaging and tissue pathology for the recurrence risk and MRD (minimal residual disease) surveillance [54]. AI pipelines like RED accelerate rare cell detecting, while ctDNA has prognostic value specially in advanced stage breast cancer, and this is being explored for earlier stage monitoring. Moreover, algorithms such as Rare Event Detection can automatically identify rare cancer cells among millions of blood cells within minutes, a task that would otherwise be labor-intensive and highly prone to human error. These advances have profound implications for personalized medicine, enabling clinicians to identify risk patterns more precisely and tailor treatments to individual biological profiles [51]. Randomized and large-scale evaluations show that AI-supported screening modalities can raise detection while keeping false positives unchanged. Also, it can cut workload as seen in the MASAI case study [46]. Decision referral and single reader replacement should be taken into consideration to assess diagnosis outcomes; ultimately accurate diagnosis will depend on a well-established workflow. A recent study by Fisches et al., 2024 [55] shows that AI does not only replicate the existing diagnosis inconsistencies among radiologists but also reduces false negative-positive recalls and missed cancers. The referenced article [55] shows that AI-integrated mammography showed close to perfect cancer detection accuracy; it correctly localized 98.1–98.9% of screen detected cancers and 87.3–93.7% when analyzed images previously read by a radiologist [55]. This supports AI ability to fill the gap where radiologists are prone to miss diagnosis. This study gives a glimpse of the root cause of false-negative and positive of traditional screening methods; it emphasizes that AI captured cancers that are prone to be missed by radiologists. For instance, when AI-system is not sure about a suspicious area in the breast, it sends for the next round screen and flags it as “suspicious” instead of immediately assigning a diagnosis, this demonstrates that AI minimizes or corrects errors that are likely to be made by a radiologist due to factors such as work fatigue, perceptual limits and bias based on experiences and these findings demonstrate that AI reduces false positives and missed cancers through mechanisms that compensate for human variability, confirming that AI does not replace human expertise but complement [55].

Collectively, these findings support the conclusion that AI integration into traditional breast cancer screening represents more than a technological enhancement; it signifies a fundamental shift in diagnostic practice. The synergistic interaction between human expertise and machine intelligence enables a hybrid model that maximizes sensitivity, specificity, and efficiency while minimizing human error. This combination shifts breast cancer diagnosis from a reactive to a proactive and patient-centered process. A distinctive contribution of this review lies in its comprehensive comparative analysis across all major breast cancer screening modalities, including mammography, ultrasound, MRI, and biopsy. It emphasizes not only the superior diagnostic performance of AI-integrated systems but also their collaborative potential to transform early detection and recurrence prediction.

Despite these advances, several limitations were evident across the reviewed studies. Many lacked external validation, limiting generalizability and increasing the risk of algorithmic bias. In some cases, sample sizes were as small as 70, while other studies lacked sufficient population heterogeneity, both of which are critical limitations when evaluating diagnostic tools in diverse clinical settings. This variation in sample populations at different diagnostic timepoints limits cross-modality comparability. Moreover, cross modality comparisons are inherently limited because they are applied at different stages of the diagnosis process. For instance, mammography is the gold standard for routine (preventive) breast cancer screening hence why in studies where AI is integrated into mammography the population may be larger compared to Ultrasound which is seen as a supplemental evaluation for women with suspicious lesions and MRI is often used for women with potential risks of developing cancer overtime time. Future research should adopt harmonized cohorts with prospective, multi-site designs to assess robustness under standardized conditions.

Furthermore, none of the reviewed AI systems have received FDA approval or have been fully integrated into routine clinical workflows. Instead, these studies primarily serve as foundational research for future integration of AI into precision diagnostics, which explains why most were retrospective in nature. Ethical concerns regarding the use of AI to process confidential patient information also remain a significant consideration. Current analyses suggest that AI-assisted traditional screening modalities such as mammography can be cost saving (up to €59k) versus double reading [56]. AI- guided risk-stratified screening protocols can yield a positive net monetary benefit at population level. They allow programs to achieve the same clinical detection outcomes with a single, targeted screening rather than requiring additional screening, and this is possible because many algorithms as said, are designed to flag suspicious areas of the breast allowing clinicians to pay closer attention to patients with signs of breast cancer. Moreover, real world costs depend on licensing and workflow [57]. In low- and middle income countries, adoption requires attention to infrastructure, training, and financing to avoid widening inequities, these requirements are contributing factors that make implementation in such region difficult [58]. It is true that the implementation based on AI-technology is challenging and AI-based cancer diagnostics systems are no different, and these are often limited by financial and infrastructure constraints [58]. Literature shows that partnership between organizations of different sectors might help mitigate these challenges, and these partnerships can happen through several pathways: mobile health, public and private partnerships between organizations in the medical field, government subsidies, device-sharing models, integration of AI tools into public health frameworks will collectively reduce operational costs and enhance accessibility in low-resources settings [59].

Across all studies included in this review, HIPAA regulations were strictly followed, and institutional review board approval was obtained with informed consent waived when appropriate. Adherence to established research guidelines remains essential to ensure that patient data is used ethically, responsibly, and with appropriate consent. Through this systematic review, we conclude that integrating AI into traditional breast cancer screening methods is effective and could potentially save hundreds of thousands of lives, although important gaps remain. Looking ahead, further research is essential across all modalities, particularly mammography, given its widespread use and affordability. Crucially, future studies must include larger and more diverse populations to train and validate AI models, thereby ensuring the generalizability and robustness of current findings. In addition, research evaluating the cost-effectiveness of integrating AI into these modalities is vital, not only for healthcare providers and hospitals but also for patients, who remain at the center of this innovation.

## CONCLUSIONS

This article establishes artificial intelligence as an indispensable ally in precision medicine, demonstrating that it does not replace human expertise but rather augments it with unparalleled analytical speed, consistency, and pattern recognition capabilities, thereby redefining diagnostic excellence.

## APPENDIX 1 - AI USE DISCLOSURE

1. AI Tool: OpenAI ChatGPT; Web Version.
2. Access Date: September 2025 to December 2025
3. Model: GPT-5.0
4. Tasks Performed:
  - Assisted with selecting and grouping articles from relevant abstracts.
  - Assisted in sections outlining
  - Assisted in editing/grammar corrections
5. Tasks AI did not perform:
  - Selection of included and excluded studies
  - Data extraction
  - Statistical analysis
6. All AI prompts and outputs were checked for factual accuracy and fully edited by the authors and all the references were verified against the original sources
